# Serotype-dependent recombinant adeno-associated vector (AAV) infection of Epstein–Barr virus-positive B-cells, towards recombinant AAV-based therapy of focal EBV + lymphoproliferative disorders

**DOI:** 10.1186/s12985-021-01695-w

**Published:** 2021-11-18

**Authors:** Elham Ahmadi, Mehrdad Ravanshad, Jun Xie, Rajesh Panigrahi, Sandeep S. Jubbal, Santosh Kumar Guru, Gao Guangping, Mazyar Ziyaeyan, Joyce Fingeroth

**Affiliations:** 1 Department of Virology, Faculty of Medical Sciences, Tarbiat Modares University, P.O. Box 14155-331, Tehran, Iran; 2 Division of Infectious Diseases and Immunology, Department of Medicine, University of Massachusetts Medical School, 364 Plantation Street, Worcester, MA 01605 USA; 3 Horae Gene Therapy Center, University of Massachusetts Medical School, 364 Plantation Street, Worcester, MA 01605 USA; 4 Department of Molecular, Cell and Cancer Biology, University of Massachusetts Medical School, 364 Plantation Street, Worcester, MA 01605 USA; 5 Alborzi Clinical Microbiology Research Center, Namazi Hospital, Shiraz University of Medical Sciences, Shiraz, Iran

**Keywords:** rAAV, EBV, B-cells, Focal tumors, PTLD, Mononucleosis, Suicide gene, Transfection

## Abstract

**Background:**

B-cell proliferative disorders, such as post-transplant lymphoproliferative disease (PTLD), are increased among persons afflicted by T-cell compromise. Most are Epstein–Barr virus (EBV) + and can first present with a focal lesion. Direct introduction of oncolytic viruses into localized tumors provides theoretical advantages over chemotherapy, immunotherapy and radiation therapy by reducing systemic toxicity. Despite extensive study as a vehicle for gene therapy, adeno-associated viruses (AAV) have rarely been applied to human cancer research due to technical and theoretical obstacles. Moreover, human B-cells have historically been described as resistant to AAV infection. Nonetheless, advances using different recombinant (r)AAV serotypes with unique tropisms to deliver cytotoxic therapy suggested a localized anti-tumor approach was feasible.

**Methods:**

As a prelude to the development of a therapeutic vehicle, the ability of fifteen distinct EGFP-bearing rAAV serotypes to transduce human B-cells, including primary, immortalized, and B-cell tumor lines ± EBV was assessed by confocal microscopy, flow cytometry and subsequently cell viability assay.

**Results:**

Rank order analysis revealed augmented transduction by rAAV6.2 and closely related virions. EBV infection of EBV-negative B-cell tumor lines and EBV immortalization of primary B-cells increased susceptibility to rAAV6.2 transduction. As a proof of concept, transduction by rAAV6.2 encoding herpes simplex virus type 1 (HSV1)-thymidine kinase (TK) eliminated TK-negative rhabdomyosarcoma cells and diminished viability of transduced B-cell lines upon incubation with ganciclovir.

**Conclusions:**

rAAV serotypes differentially transduce human B-cell lines reversing the dogma that human B-cells are refractory to AAV infection. EBV + B-cells display increased susceptibility to rAAV6.2 infection, uncovering a new method for improved nucleic acid transfer into transfection-resistant B-cell lines. The introduction of a functional suicide gene into the rAAV6.2 genome identifies a candidate vector for the development of rAAV-based oncolytic therapy targeting focal EBV-bearing B-lymphoproliferative disorders.

## Background

B-cell proliferative disorders, such as post-transplant lymphoproliferative disease (PTLD) and certain B-cell lymphomas, occur with increased frequency among persons with T-cell compromise. These disorders are primarily Epstein–Barr Virus (EBV) + and can first present with a focal lesion accessible to direct inoculation of therapeutic agents [4, 7]. Target tissues frequently include Waldeyer's tonsillar ring, B-cell-associated lymphoid tissues of transplanted organs, solitary lymph nodes, and central nervous system lesions. Direct introduction of oncolytic viruses into localized tumors provides theoretical advantages over chemotherapy, immunotherapy and radiation therapy by reducing systemic toxicity to which the immunocompromised host is especially vulnerable [8].

Although widely studied as a safe vehicle for gene therapy, adeno-associated viruses (AAV) have infrequently been applied to treat cancers [14]. Moreover, early work indicated human B-cells were poor targets for AAV [9, 16, 26]. Consequently, little is known about AAV infection of human B-cell tumors, despite widespread derivation in recent years of AAV recombinants (rAAV) with altered tropisms [5, 25].

Unlike oncolytic viruses, recombinant (r)AAV is nonpathogenic, does not independently replicate nor integrate into host cell DNA. The rAAV genome is eventually eliminated, reducing the risk of inadvertent spread to normal cells. Genes, including gene regulatory products engineered to eliminate only tumor cells, can be introduced into the AAV genome [20]. Although an immune response to AAV-infected cells can impede gene therapy, this may prove advantageous in the setting of short-term anti-tumor therapy [21], as often required for the spectrum of PTLD-associated tumors.

Herein, we examined and compared the in vitro ability of fifteen select recombinants derived from a well-characterized self-complementary (sc)rAAV plasmid vector that encodes enhanced green fluorescent protein (EGFP) [16] to transduce human B-cells. The focus was on B-cells infected and immortalized with EBV. A rank order analysis of serotypes revealed rAAV6.2 transduction consistently supported EGFP expression in human B-cells and that the percentage of fluorescing cells was highest among B-cells bearing EBV. When herpes simplex virus type 1 (HSV1)-thymidine kinase (TK) was introduced into the rAAV6.2 genome, transduced human TK negative rhabdomyosarcoma cells were eliminated upon exposure to ganciclovir, and the viability of transduced B-cell tumor cells was reduced, providing support for development of rAAV-based oncolytic therapy. This proof-of-concept study demonstrates AAV serotypes such as rAAV6.2 preferentially transduce human EBV + B-cells and can support the expression of suicide genes, highlighting the potential for developing selective rAAV-mediated treatment of focal EBV + proliferative disorders.

## Methods

### Source of rAAV virions

Fifteen distinct serotypes of a single self-complementary rAAV-EGFP plasmid vector [28] were produced by differential expression of capsid proteins at the Horae Gene Therapy Center of the University of Massachusetts Medical School. The parent rAAV-EGFP plasmid vector encodes a cytoplasmic enhanced green fluorescent protein (EGFP) driven by a hybrid CMV enhancer/chicken β-actin promoter [27]. Following transduction, EGFP fluorescence can be directly visualized in cells by microscopy (confocal microscopy, flow cytometry) and protein expression can be detected with specific antibodies (see below). Each of the fifteen rAAV serotypes (Table 1) were synthesized in modified human HEK293 cells, extracted, purified, isolated, analyzed for genome content and titered as described [25]. Production details for each of the fifteen serotypes are available through the Horae Gene Therapy Center.

**Table 1:** rAAV EGFP Serotypes

rAAV EGFP serotypes	Titer (GC/mL)
AAV1	1.8E + 13
AAV2	1.0E + 12
AAV3b	6.0E + 12
AAV4	1.2E + 13
AAV5	1.4E + 13
AAV6	8.0E + 12
AAV6.2	8.0E + 12
AAV6TM	8.0E + 12
AAV7	1.5E + 12
AAV8	7.0E + 12
AAV9	2.0E + 13
AAVrh8	8.0E + 12
AAVrh10	8.0E + 12
AAVrh39	1.0E + 13
AAVrh43	6.0E + 12

Fifteen distinct serotypes of rAAV, including four serotypes derived from rhesus monkeys (rh) were obtained according to procedures developed in the Horae Gene Therapy Center at the University of Massachusetts Medical School (Methods). Virions were produced in HEK293 cells (Methods). Virus titer was expressed as genome copies per ml (GC/ml). Titers were subsequently normalized for comparative transduction assays

### Source of cells

B-cell lines used in this study are listed in Table 2 together with their source. Primary B-cells were isolated from human spleen samples obtained from the New England Organ Bank in accordance with the policies of the Institutional Review Boards of the NEOB and of the University of Massachusetts Medical School. Primary B-cell isolation was performed by negative selection using the EasySep Direct Human B Cell Isolation Kit (STEMCELL Technologies) according to the manufacturer’s directions. Greater than 90% purity of the B-cell population was verified by flow cytometry using the monoclonal antibodies: APC-conjugated anti-CD20 (BioLegend) and FITC-conjugated anti-CD19 (BioLegend). All B-cells were maintained in RPMI-1640 (Sigma-Aldrich) supplemented with 10% heat-inactivated fetal calf serum (HyClone), 100 U/ml penicillin, and 100 μg/ml streptomycin (Cellgro) at 37 °C in a 5% CO_2_ incubator.

**Table 2:** Sources of Human B-cells

Cell type	Source	Description
*Primary cells*		
Primary B-cell EBV-	New England Organ Bank	Human spleen
*B-LCL*		
Newly EBV-infected primary B-cell	In vitro infection with B95-8 virus strain	Human spleen
*Standard B-lymphoblastoid cell line*		
B95-8	ATCC-CRL-1612	Marmoset B-cells, immortalized with the prototype B95-8 EBV strain
*EBV + Burkitt lymphoma lines*		
P3HR1	ATCC-HTB-62	Burkitt lymphoma derived from Jijoye Burkitt lymphoma
Daudi	ATCC-CCL-213	Burkitt lymphoma
Raji	ATCC-CCL-86	Burkitt lymphoma
*EBV-Burkitt lymphoma lines*		
Ramos	ATCC CRL-1596	Burkitt lymphoma
BL41	ATCC-ACC160	Burkitt lymphoma
*In vitro-infected EBV + Burkitt’s lymphoma lines*		
Ramos/B95-8	Fred Wang and Elliot Kieff Harvard University	In vitro EBV-infected Burkitt lymphoma line
BL41/B95-8	Fred Wang and Elliot Kieff Harvard University	In vitro EBV-infected Burkitt lymphoma line

Cell lines included two EBV−, five EBV+ Burkitt lymphoma lines, and two EBV-immortalized lymphoblastoid cell lines (LCLs), as well as primary B-cells

*ATCC* American Type Culture Collection (Manassas, Virginia)

### Transduction procedures and confocal microscopy

To compare the transduction efficiency of each of the fifteen rAAV serotypes in each of the human B-cell lines, cells grown to mid-log phase were seeded in 24-well plates at a density of 2 × 10^5^ cells per well. Twenty-four hours later, cells were transduced with an rAAV serotype at an MOI of 10^5^. Forty-eight hours post-infection, fluorescence mediated by EGFP expression was captured by a ZEISS LSM 700 confocal microscope.

### Transduction procedures and flow cytometry

B-cells from each of the described sources (Table 2) were grown to mid-log phase and then plated (3 × 10^5^ cells/well) in 24-well plates. The cells were then transduced with each of the 15 rAAV serotypes at an MOI of 10^5^ in independent experiments that were repeated in triplicate, n = 3. At 48 h post-infection, the cells were harvested, washed with PBS, fixed with 2% paraformaldehyde and analyzed by flow cytometry on an LSRII flow cytometer (BD Biosciences). The BD FACSDiva™ Software (BD Biosciences) was used to quantify the percentage of fluorescent (EGFP-expressing) cells.

### Synthesis and production of rAAV6.2 encoding HSV1-TK

The rAAV-based plasmids encoding EGFP-HSV1-TK and HSV1-TK alone were synthesized by Gene Universal (Newark, Delaware). The respective genomes are displayed in Fig. 4a. The source of the inserts (EGFP-HSV1-TK and HSV1-TK) was previously described [6]. Each genome was encapsidated to yield the rAAV6.2 serotype and produced for transduction experiments in the Horae Gene Therapy Center as described above.

The 143BTK- human rhabdomyosarcoma cell line, which lacks human TK-1 expression (ATCC), was pre-selected for bromodeoxyuridine resistance. Cells were maintained in DMEM (Corning) supplemented with 10% heat-inactivated fetal calf serum (HyClone), 100 U/ml penicillin, and 100 μg/ml streptomycin (Cellgro) at 37 °C in a 5% CO_2_ incubator. 143BTK cells were plated and transduced with either rAAV6.2-EGFP-HSV1-TK or rAAV6.2 HSV1-TK as described above for confocal analysis of B-cell lines.

### Immunoblot confirmation of HSV1-TK protein expression in transduced cells

Selected B-cell lines grown to mid-log phase were seeded in 24-well plates at a density of 2 × 10^5^ cells per well. Twenty-four hours later, cells were transduced with an rAAV6.2 serotype encoding HSV1-TK at an MOI of 10^5^. Forty-eight hours after transduction, cells were harvested, lysed in RIPA buffer and quantified by Bradford assay. Total cellular protein (20 µg per lane) was separated by NuPAGE Bis–Tris (4–12%) polyacrylamide gel electrophoresis (Thermo Fisher Scientific) in MOPS buffer under reducing conditions. Protein was transferred onto a nitrocellulose membrane that was blocked with 5% skimmed milk in TBST for one hour prior to antibody incubation, five membrane washes and final detection with the ECL chemiluminescent detection kit, Clarity Max Western ECL Substrate (Bio-Rad).

The primary antibody rabbit polyclonal anti-HSV1-TK was generously provided by Dr. William Summers of Yale University. HRP-labeled goat anti-rabbit secondary antibody was used for final detection (Santa Cruz Biotechnology).

The primary antibodies, mouse anti-EGFP and mouse anti-GAPDH, were obtained from Santa Cruz Biotechnology, as were HRP-labeled goat anti-mouse IgG secondary antibodies used for chemiluminescent detection.

### Sensitivity of HSV1‐TK‐transduced cells to GCV

Confocal images of 143BTK- cells transduced with rAAV6.2 HSV1-TK or with EGFP-HSV1-TK were collected using a ZEISS LSM 700 confocal microscope. Pictures were analyzed using the ZEN lite software. All pictures were taken at the same magnification (scale bar = 50 μm). Images were collected before and then 72 h after incubation with 10 μM ganciclovir. The concentration of ganciclovir displayed was established by a dose–response curve during prior experiments.

### MTT viability analysis of rAAV6.2 HSV1-TK transduced B-cells incubated with ganciclovir

Selected B-cell lines (2 × 10^5^ cells/ml) were cultured in 96 well microplates for 24 h at 37 °C. Cells were then transduced by rAAV6.2-HSV1-TK for 48 h. Transduced cells were incubated with 10 μM GCV or placebo (no GCV) for three days. After visualization by microscopy, twenty microliters of MTT (3-(4,5-dimethylthiazol-2-yl)-2,5-diphenyltetrazolium bromide) solution (5 mg/ml) was added to each well and incubated with the cells for four hours. The accumulated formazan product was solubilized by adding 40 μl of DMSO. The optical absorption of the reaction product was measured at 570 nm. The percentage of surviving cells was calculated by measuring the mean absorbance of treated cells over the mean absorbance of untreated cells. Untreated cells were 100% viable. Results were represented as mean ± SD (n = 3).

### Statistical analysis

Statistical analysis of fluorescence data generated by flow cytometry was performed with GraphPad Prism 8 software (GraphPad, San Diego, CA). Because there was minimal variation within conditions, we assumed all data followed a normal distribution. Values from independent experiments were reported as mean ± SD.

### Ethical approval

This study was performed in accordance with the policies of the Institutional Review Boards of the NEOB and of the University of Massachusetts Medical School (IRB #H00004283).

## Results

### Examination of ten human B-cell lines ± EBV infection with fifteen distinct recombinant self-complementary adeno-associated viral vectors

Although primary human B-cells have been described as poor targets for founder AAV vectors [17], little is known about their susceptibility to infection by rAAV serotypes that display novel tropisms. Even less is known about the susceptibility of human B-cell tumor lines to the spectrum of rAAV serotypes. To address the potential of rAAV vectors to mediate anti-tumor therapy, fifteen distinct rAAV candidates [25] encoding EGFP were analyzed (Table 1) for their ability to infect ten human B-cell lines. These lines included two EBV-, five EBV + Burkitt lymphoma lines, and two EBV-immortalized lymphoblastoid cell lines (LCLs), as well as primary B-cells (Table 2).

**Fig. 1:**
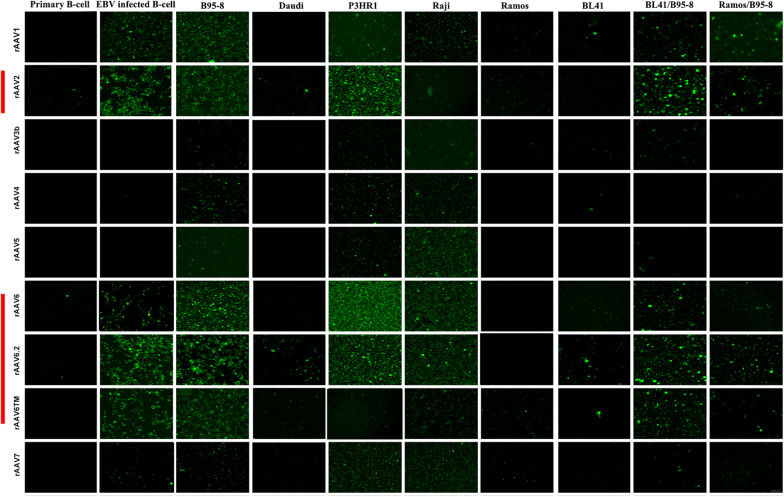
Visualization of EGFP fluorescing B-cells following rAAV transduction. Human B-cells derived from ten distinct sources (Table 2) were individually transduced with each of the fifteen rAAV serotypes encoding EGFP as displayed in Table 1. Transduction of cells was performed using standard procedures at a MOI of 10^5^ (Methods). Fluorescent cells were visualized 48 h after transduction by confocal microscopy (Methods). The nine rAAV serotypes that produced B-cell fluorescence are displayed. The serotypes that produced the highest percentage of fluorescent cells across all B-cell sources are indicated by a vertical red line in the left margin of the figure

Upon titer normalization (10^5^ genome copies/ml), each of the rAAV serotypes listed in Table 1 was independently transduced into each of the human B-cell sources under study (Table 2). Detection of EGFP fluorescence in the respective cellular targets was initially assessed by confocal microscopy (Fig. 1). Although variation in fluorescence was observed in relation to the rAAV vector of origin as well as the target B-cell (Fig. 1), overall, the rAAV6.2 serotype resulted in the highest percentage of B-cells that expressed EGFP irrespective of the cell line source. Similar patterns of fluorescence were noted among the serotypes most closely related to 6.2 (AAV6, AAV6TM, AAV2) as indicated by a vertical red line in the right margin of Fig. 1.

### Tumor and immortalized B-cells were more susceptible to rAAV6.2 transduction than primary B-cells

**Fig. 2:**
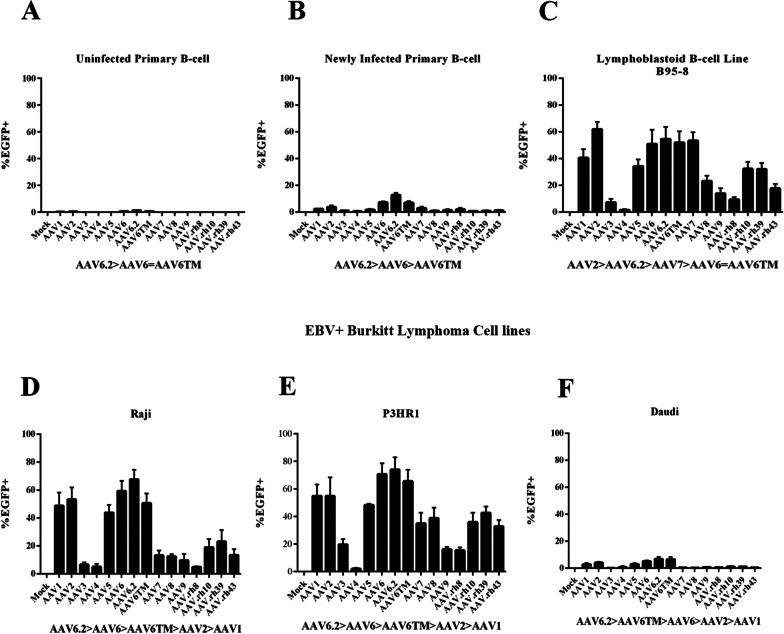
Flow cytometric analysis of EGFP fluorescing B-cells following rAAV transduction. **a**–**c** Bar graphs demonstrating the percentage of EGFP-expressing cells (y-axis) following transduction of different rAAVs (x-axis) as detected by flow cytometry in **a** uninfected primary B-cells, **b** primary B-cells 48 h after EBV infection, and **c** B-cells stably immortalized by the prototype EBV B95-8 virus, **d–f** Bar graphs demonstrate the percentage of EGFP-expressing cells (y-axis) detected in three different EBV + Burkitt lymphoma cell lines: **d** Raji, **e** P3HR1, and **f** Daudi as assessed by flow cytometry 48 h after transduction of different rAAV serotypes (x-axis). The EBV genomes of the respective tumor lines vary based on mutation, deletion and inversions. The bar graphs display mean ± SD, n = 3 independent experiments. A rank order of the serotypes producing the highest percentage of fluorescent cells is displayed under each histogram

To quantitate percent EGFP fluorescence in primary cells compared to immortalized and tumor cell lines and to more precisely determine whether EBV infection altered EGFP detection, transduction studies were next performed using flow cytometry for analysis (Fig. 2a–f). EGFP fluorescence in primary B-cells was minimal irrespective of the transduced rAAV serotype, consistent with previous reports that founder AAV vectors do not infect normal human B-cells (Fig. 2a). A modest increase in EGFP was detected 48 h after EBV infection of primary cells (1–10%) (Fig. 2b). Fluorescence increased to ~ 50% of cells in the prototype EBV immortalized cell line B95-8 after transduction of rAAV6.2 and closely related serotypes (Fig. 2c). Infection of three naturally occurring EBV + Burkitt lymphoma lines revealed EGFP expression in each line with rAAV6.2 infection yielding the highest percentage of EGFP-producing cells per line. However, whereas the percent of fluorescent cells was 60–80% following rAAV6.2 transduction of Raji (Fig. 2d) and P3HR1 (Fig. 2e), only ~ 7% of cells revealed detectable EGFP in Daudi (Fig. 2f). It is known from prior DNA sequence analysis (NCBI) that each of these EBV + tumor lines contains distinct deletions and mutations relative to the prototype B95-8 genome. Clones of Daudi, in particular, have been shown to vary in the expression of the major EBV protein LMP1 [11]. As EBV products promote specific phenotypic changes in infected cells [2], comparative analysis of the respective genomes may uncover B-cell modifications associated with efficient rAAV6.2 transduction, as discussed below.

### EBV “superinfection” of EBV-negative Burkitt tumor lines increased susceptibility to rAAV6.2

**Fig. 3:**
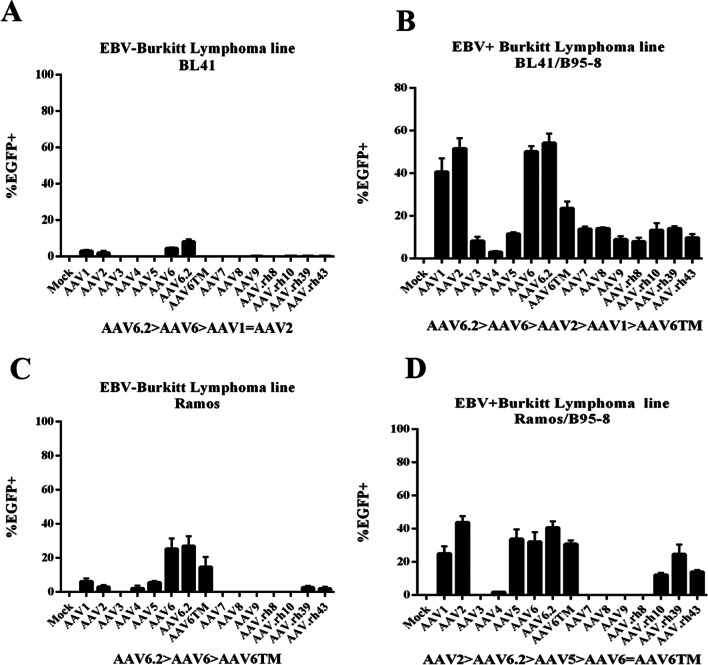
EBV “superinfection” increases efficiency of rAAV transduction. Percentage of EGFP-expressing cells (y-axis) in Burkitt lymphoma cell lines in the absence or presence of the B95-8 EBV genome was detected by flow cytometry 48 h after transduction of distinct rAAV serotypes (x-axis). Top (**a**, **b**), comparison of BL41 and BL41-B95-8. Bottom (**c**, **d**), comparison of Ramos and Ramos/B95-8. The bar graphs display mean ± SD, n = 3 independent experiments. A rank order of the serotypes producing the highest percentage of EGFP + cells is displayed under each histogram

To further address whether EBV infection facilitates B-cell transduction by rAAV6.2, two EBV-negative Burkitt lymphoma lines BL41 and Ramos that are stably “superinfected” with the EBV B95-8 strain were transduced with each of the fifteen rAAVs described in Table 1. Superinfection with the B95-8 prototype virus is known to elicit phenotypic changes in EBV-negative Burkitt lines. In fact, BL41/B95-8 was previously shown to display phenotypic changes typical of those that occur upon antigen activation of primary B-cells [2]. After transduction of rAAV6.2 (and related serotypes), the percentage of fluorescing cells in BL41 compared with BL41/B95-8 increased from less than 10% to greater than 50% (Fig. 3a, b). EGFP expression after AAV6.2 infection of Ramos/B95-8 was likewise increased (Fig. 3c, d) though the difference was less than in the BL41-derived line.

### rAAV6.2 encoding HSV1-TK transduced tumor cells were eliminated upon incubation with ganciclovir

**Fig. 4:**
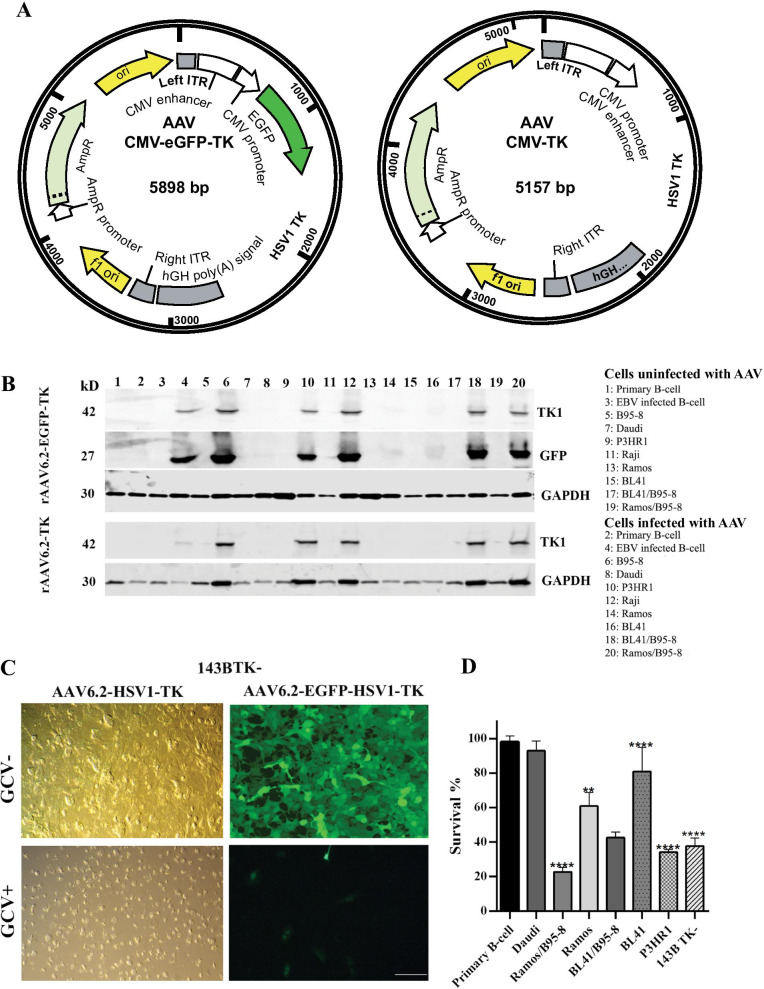
Evaluation of rAAV-CMV-EGFP-HSV1-TK and rAAV-CMV-HSV1-TK in vitro. **a** Diagrammatic representation of rAAV6.2 expressing HSV1-TK. rAAV-CMV-EGFP-HSV1-TK (left) and rAAV-CMV-HSV1-TK (right) plasmids were constructed as described (Methods). rAAV6.2 expressing the respective plasmids was produced by the Horae Gene Therapy Center. **b** Detection of EGFP and HSV1-TK proteins by immunoblot. rAAV6.2 bearing EGFP-HSV1-TK (top) or HSV1-TK alone (bottom) versus no virus control was used to infect the indicated B-cell lines as listed in the right margin GAPDH was used as a loading control. EGFP and HSV1-TK were coordinately expressed (top) when rAAV6.2-EGFP-TK was transduced. The pattern of TK1 expression was unchanged when rAAV6.2TK1 alone was transduced. The quantity of EGFP protein detected in each line by immunoblot paralleled the relative fluorescence of the same lines (Figs. 1, 2), highlighting the reproducibility of rAAV6.2 transduction. **c** Functionality of rAAV6.2 encoding HSV1-TK. 143B TK cells transduced with either rAAV6.2 HSV1-TK or rAAV6.2 EGFP-HSV1-TK (top panels) were visualized by confocal microscopy. Cells were next incubated with 10uM ganciclovir for 72 h (bottom panels) and again visualized. The bottom left panel shows that only rounded and dying cells remain. The bottom right panel confirms that virtually all fluorescent cells have been eliminated. **d** Loss of viability among rAAV6.2 HSV1-TK transduced cells incubated with ganciclovir. Representative B-cell lines and 143B TK-cells (control) were transduced with rAAV6.2-HSV1-TK. Twenty-four hours later cells were incubated with 10uM ganciclovir for 72 h at which time an MTT-based assay was performed to measure cell viability. Results are displayed as percent survival (y-axis) of representative B-cell lines (x-axis). Inverse correlation of viability with percentage fluorescence of the identical cell lines displayed in Figs. 1, 2 underscores the reproducibility of rAAV6.2 transduction of specific B-cell lines

Treatment of EBV PTLD typically involves exposure to systemic agents that can compromise the survival of transplant recipients and are required even when PTLD presents as a localized growth. Thus, precision approaches are needed. Expression of HSV1-TK in cells specifically converts the nucleoside analog ganciclovir to ganciclovir triphosphate, promoting nucleotide incorporation into cellular DNA and apoptosis [12]. The subsequent release of ganciclovir triphosphate into the tumor microenvironment results in preferential uptake by actively proliferating adjacent tumor cells causing bystander killing [19]. These findings suggested rAAV serotypes, such as rAAV6.2 bearing HSV1-TK, could contribute to eliminating B-cell tumor cells upon direct injection [18] of rAAV into lesions. Although HSV1-TK potently phosphorylates ganciclovir, nucleoside kinases that are activated in rapidly growing tumor cells can also phosphorylate ganciclovir, though to lesser degrees. First, to confirm the ability of rAAV6.2 encoding HSV1-TK to eliminate transduced tumor cells, the prototype TK negative human rhabdomyosarcoma cell line, 143B TK- [22] followed by a subset of B-cell lines ± EBV were evaluated for response to ganciclovir. This was achieved through sequential experiments, including modification of the rAAV6.2 genome to express fused EGFP-HSV1-TK (Fig. 4a, left-hand side) or HSV1-TK alone (Fig. 4a, right hand side). Infection was demonstrated by detection of EGFP-HSV1-TK and HSV1-TK protein in transduced cells by immunoblot and (Fig. 4b) by selective elimination of transduced HSV1-TK expressing cells in the presence of ganciclovir.

A diagrammatic representation of the rAAV6.2 genomes modified to express either a fusion of EGFP-HSV1-TK or HSV1-TK alone is displayed in Fig. 4a. Co-expression of EGFP and HSV1-TK, as well as unique expression of HSV1-TK, was confirmed upon immunoblot of transduced cell lines (Fig. 4b). Of note, there was a quantitative correlation between cell lines that displayed the highest percentage of EGFP fluorescence in Figs. 1 and 2 and those in which HSV1-TK protein was most abundantly detected (Fig. 4b), underscoring the reproducibility of serotype transduction. Confocal analysis (Fig. 4c) demonstrated that incubation of 143B TK negative cells transduced by AAV6.2 EGFP-HSV1-TK (*left panels*) or AAV6.2 HSV1-TK (*right panels*) with ganciclovir resulted in the elimination of most 143B TK-expressing cells, confirming the functionality of HSV1-TK. Transduction of AAV6.2 HSV1-TK into representative B-cell lines incubated with ganciclovir likewise resulted in loss of cell viability as measured by the tetrazolium dye MTT 3-(4,5-dimethylthiazol-2-yl)-2,5-diphenyltetrazolium bromide (MTT) assay (Fig. 4d). The comparative loss of viability among EBV ± B-cell populations assayed by MTT (Fig. 4d) inversely correlated with the percentage of fluorescent cells following rAAV6.2 EGFP transduction (Figs. 1 and 2), again underscoring the reproducibility of transduction mediated by rAAV6.2.

## Discussion

Among the most devastating consequences of successful hematologic and solid organ transplant is the development of PTLD. PTLD is a consequence of T-cell suppression required for engraftment of foreign cells and primarily results in uncontrolled outgrowth of EBV-bearing B-cells that run the gamut from normal appearing activated lymphocytes to aggressive clonal B-cell malignancies. Persons with other causes of T-cell deficiency (iatrogenic, AIDS, congenital diseases) are also at increased risk of these EBV-associated disorders. Rarely, PTLD can present as an EBV + T-cell outgrowth or an EBV + leiomyosarcoma. While aggressive surgery, chemotherapy, immunotherapy and radiation therapy variably control disease, therapy is often poorly tolerated by persons who are already profoundly immunosuppressed. PTLD may originate as a local growth—in Waldeyer’s ring, especially after a primary infection, in the B-cell associated infiltrate of an allograft, or as an isolated lymph node or a CNS lesion. Focused therapy that precisely targets individual lesions could significantly reduce treatment-based morbidity and mortality.

Among the severe manifestations of primary EBV infection is acute infectious mononucleosis (AIM) complicated by airway obstruction. This is caused by the exuberant proliferation of lymphocytes in the vicinity of Waldeyer’s ring. High-dose steroid therapy, while often effective, eliminates not only the EBV + B-cells but also other immune cells required to resolve the infection and thereby causes a clinical conundrum. rAAV administration raises the prospect of focused, effective therapy.

AAV as a therapeutic modality has been predominantly associated with gene replacement. In recent years, multiple innovations affecting the structure and content of the viral genome, the ability to alter and re-direct tropism by capsid mutation as well as utilization of new techniques that augment manufacturing [25] has sparked interest in applying rAAVs to precision cancer therapy – systemic and targeted [14]. Because initial studies indicated human B-cells were not susceptible to AAV infection, little published information was available relevant to cancers of B-cell origin. Nevertheless, current innovations suggested application of new rAAV variants to the treatment of B-cell tumors with emphasis on focal lesions amenable to direct introduction of comparatively small quantities of virus, would be valuable.

Results of the current proof-of-concept analysis revealed that among the fifteen rAAV serotypes evaluated, rAAV6.2 and those rAAVs closely related to 6.2 (rAAV6, rAAV6TM), based on capsid sequence, were most efficient in virus transduction. All fifteen rAAV candidates contained identical EGFP-encoding genomes, implicating differential encapsidation as the likely source of altered transduction efficiency. Although the percentage of cells transduced by rAAV6.2 varied between B-cell sources, transduction efficiency was highly reproducible in different assays and even when genome content was altered by the introduction of HSV1-TK. Primary B-cells were minimally transduced by all fifteen rAAV serotypes, though rAAV6.2 was most effective on a comparative basis. This result was entirely consistent with older observations that primary B-cells were not viable targets for AAV transduction. In contrast, B-cells that contained intact EBV genomes were among the most effectively transduced cells. Infection of primary B-cells and EBV negative cell lines by the prototype EBV B95-8 virus uniformly increased their susceptibility to rAAV6.2 transduction.

In addition to demonstrating the utility of rAAV6.2 as a therapeutic, these findings highlight the potential of this rAAV serotype to introduce nucleic acids into difficult to transfect human B-cell tumor lines to uncover oncogenic mechanisms. Optimization of transfection efficiency for each of the respective lines could be individually achieved.

The latent cycle EBV-encoded proteins, latent membrane proteins LMP-1 and LMP-2, mediate B-cell transformation upon B-cell activation of two main pathways, one that mimics T-cell stimulation of B-cells via CD40 and IL-4 (LMP-1), the other by mimicry of IgM receptor signaling (LMP-2) [3, 10]. As a consequence, many B-cell surface antigens present at low levels on primary resting B-cells are upregulated or exposed, potentially increasing the access of rAAV6.2. While limited in number, studies by two groups lend weight to the hypothesis that a related activation event is key. Serial experiments conducted by the Hallek laboratory (2002–2004) using rAAV2 to transduce B-CLL cells showed that pre-incubating these cells with complexed CD40L, with anti-IgM (and to a lesser extent with CpG oligos) augmented rAAV2 transduction [26]. Consistent with these observations, in 2018, Hung et al. showed that incubating primary human B-cells with a “B-cell activation cocktail” consisting of CD40L trimers, CpG and the interleukins-2, 10, 15 augmented rAAV6 transduction [9]. Comparative analysis of eight rAAVs encoding EGFP (rAAV1, 2, 2.5, 5, 6, 8, 9, D–J), conducted by Hung et al. showed rAAV6 transduced 40% of cells, rAAV2 30%, and all others < 10%. Taken together, these disparate observations highlight a key role for CD40 signaling in regulating rAAV6.2-mediated B-cell transduction (though other activation signals may also contribute). The precise mechanism through which CD40 ligation augments subsequent transduction by these serotypes remains unknown. Modulation of a specific B-cell surface receptor (upregulation, altered access) or possibly an intracellular transit pathway is suggested on the basis of capsid specificity.

What does this mean for capsid specificity? Capsid sequence variation typically occurs on the external surface in hypervariable regions that comprise approximately 19% of the overall protein and determine tropism [24]. Although tropism primarily reflects attachment to a cellular receptor(s), it can also comprise post-entry events that affect intracellular capsid stability, transport and nuclear delivery. While rAAV6.2 transduction was most efficient, the closely related serotypes rAAV6 and rAAV6TM capsids (clade A) were also effective. The AAV6.2 serotype was created by mutating the phenylalanine (F) residue at position 129 in the VP1 protein of AAV6 to leucine (L) and had also been found to increase transduction of other human cell types, such as human airway epithelium [15]. To date, the precise mechanism by which transduction is enhanced is not known. The rAVV6TM (TM = triple mutant) capsid contains three mutations Y731F/Y705F/T492V that remove residues implicated in phosphorylation-dependent proteasomal degradation, an event with potential to impede nuclear delivery [23]. However, the 6TM mutated capsid was not more efficient at human B-cell transduction. Similar to older studies, rAAV2 (clade B) ranked next in human B-cell transduction efficiency, though once again the mechanism remains unresolved.

The incorporation of HSV1-TK into the rAAV6.2 vector genome and successful demonstration of its activity post-transduction paves the way for exploration of additional suicide effectors as well as select EBV promoter/enhancer elements (e.g., EBV nuclear antigen-1, EBNA-1) that drive suicide effectors in a manner that guarantees expression only in targeted EBV + B-cell tumors [13]. The observation that primary resting B-cells are not transduced is a plus. However, because rAAV6.2 has been shown to transduce other human cells, including certain cancers, a demonstration of selective tropism will be required for clinical translation. Further mutational analysis of the rAAV6.2 capsid to identify amino acid substitutions that confer selective tropism will, therefore, be necessary. Libraries of singleton mutations in the hypervariable region of the rAAV6 capsid are available for initial screening [24], but multiple exchanges may be required. An approach that combines both vector and capsid alterations to create a highly selective rAAV6.2 variant has the potential to transform the treatment of localized EBV disease.

While development of a suitable in vivo model of focal EBV disease in rodents would confirm these results (despite caveats concerning EBV-rodent models) [1], the observation that multiple murine cell types are susceptible to rAAV6.2 [23] makes such an endeavor impracticable at this time. As discussed above, the development of a highly EBV-specific promoter able to activate a suicide gene or capsid mutations that produce exquisite cell specificity will be required.

## Conclusions

The dogma that human B-cells cannot be transduced by AAV is upset. Specific rAAVs are able to transduce human B-cells, especially when activated in the form of tumor cell lines. EBV infection of B-cells increases susceptibility to transduction by specific rAAVs. rAAV6.2 displayed maximal transduction efficiency among the 15 serotypes examined in this study, establishing the utility of rAAV6.2 and related serotypes for transduction of human B-cell lines that are often difficult to transfect. Vector support of suicide gene (HSV1-TK) expression and demonstration of subsequent cell killing provides a preliminary basis for development of rAAVs that, because of their unique properties, could eradicate focal forms of PTLD and related lesions limiting the need for toxic therapies.

## Data Availability

The datasets used and/or analyzed during the current study are available from the corresponding author and first author on reasonable request.

## Abbreviations

PTLD: Post-transplant lymphoproliferative disease
AAV: Adeno-associated virus
rAAV: Recombinant adeno-associated virus
scAAV: Self-complementary adeno-associated virus
EBV: Epstein–Barr virus
HSV1: Herpes simplex virus type 1
TK: Thymidine kinase
IM: Infectious mononucleosis
EBNA-1: Epstein–Barr virus nuclear antigen 1
LMP-1: Latent membrane protein-1
LMP-2: Latent membrane protein-2
CNS: Central nervous system
EGFP: Enhanced green fluorescent protein
LCLs: Lymphoblastoid cell lines
